# Ferroelectric Catalysts in the Hydrogen Evolution Reaction: A Perspective

**DOI:** 10.1002/advs.76739

**Published:** 2026-07-27

**Authors:** Rongxuan Lu, Weizhen Meng, Zhenxiang Cheng, Judy N. Hart, Michael Ferry, Sean Li, Wenxian Li

**Affiliations:** ^1^ School of Chemical Engineering UNSW Sydney NSW Australia; ^2^ College of Physics Hebei Key Laboratory of Photophysics Research and Application Hebei Normal University Shijiazhuang China; ^3^ Institute for Superconducting and Electronic Materials (ISEM) University of Wollongong Wollongong NSW Australia; ^4^ School of Materials Science and Engineering UNSW Sydney NSW Australia; ^5^ The ARC Centre of Excellence for Carbon Science and Innovation UNSW Sydney NSW Australia

**Keywords:** electrocatalysis, ferroelectric catalysts, HER, photocatalysis, polarization switching

## Abstract

Hydrogen, as a clean and sustainable energy carrier, is currently predominantly produced by electrocatalytic water splitting, which relies on traditional precious‐metal‐based catalysts. The use of non‐precious‐metal‐based catalysts to promote water splitting driven by renewable energy is gradually gaining favor. Recently, ferroelectric (FE) materials have attracted extensive attention in various catalytic reactions owing to their spontaneous polarization. As a model system with relatively simple reaction pathways, the hydrogen evolution reaction (HER) facilitates a deeper understanding of the role of polarization in catalytic mechanisms. This perspective highlights recent advances and conceptual developments in FE catalysts for the HER. First, we introduce the fundamental mechanisms of the HER and ferroelectricity. Second, based on structural design, FE catalysts are classified into single‐phase FE catalysts, single‐atom modified FE catalysts, FE heterostructure catalysts, and other engineered FE catalysts, and examples of research on each class of FE catalyst for the HER are discussed. Finally, prospects for the future development of FE catalysts for the HER are discussed from the perspectives of intrinsic FE metals, unconventional FE systems, and the coupling of spin with other physical properties. We hope this perspective will provide new insights and research references for exploring FE catalysts for the HER.

## Graphical abstract

This perspective summarizes recent advances in ferroelectric (FE) catalysts for the hydrogen evolution reaction (HER). It first introduces HER fundamentals and FE mechanisms, then classifies FE catalysts into single‐phase, single‐atom‐modified, heterostructured, and other engineered types, with representative examples. Finally, it outlines future directions including FE metals, unconventional FE systems, and the coupling of spin and other physical properties, aiming to inspire new insights into advanced FE catalysts.

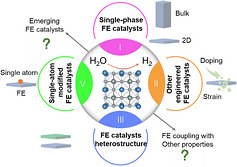

## 1 Introduction

### 1.1 Advances in Green H_2_ Production: Catalytic Pathways and Material Innovations

The advent of fossil fuels has significantly accelerated global development, yet it has also caused severe environmental damage. Meanwhile, as a finite and non‐renewable resource, fossil fuels are being rapidly depleted [1, 2]. Consequently, the development of green, renewable energy sources and the attainment of zero carbon emissions have emerged as tasks of paramount importance. The production of hydrogen (H_2_) by water splitting has recently gained considerable attention in the research community [1, 2, 3, 4]. However, industrial‐scale H_2_ generation still requires substantial amounts of electricity, which largely relies on conventional fossil fuels. This poses a significant challenge to the Sustainable Development Goals. Furthermore, current H_2_ production processes rely on traditional precious metals (e.g., Pt and Pd) as their primary catalytic materials [5].

**FIGURE 1:**
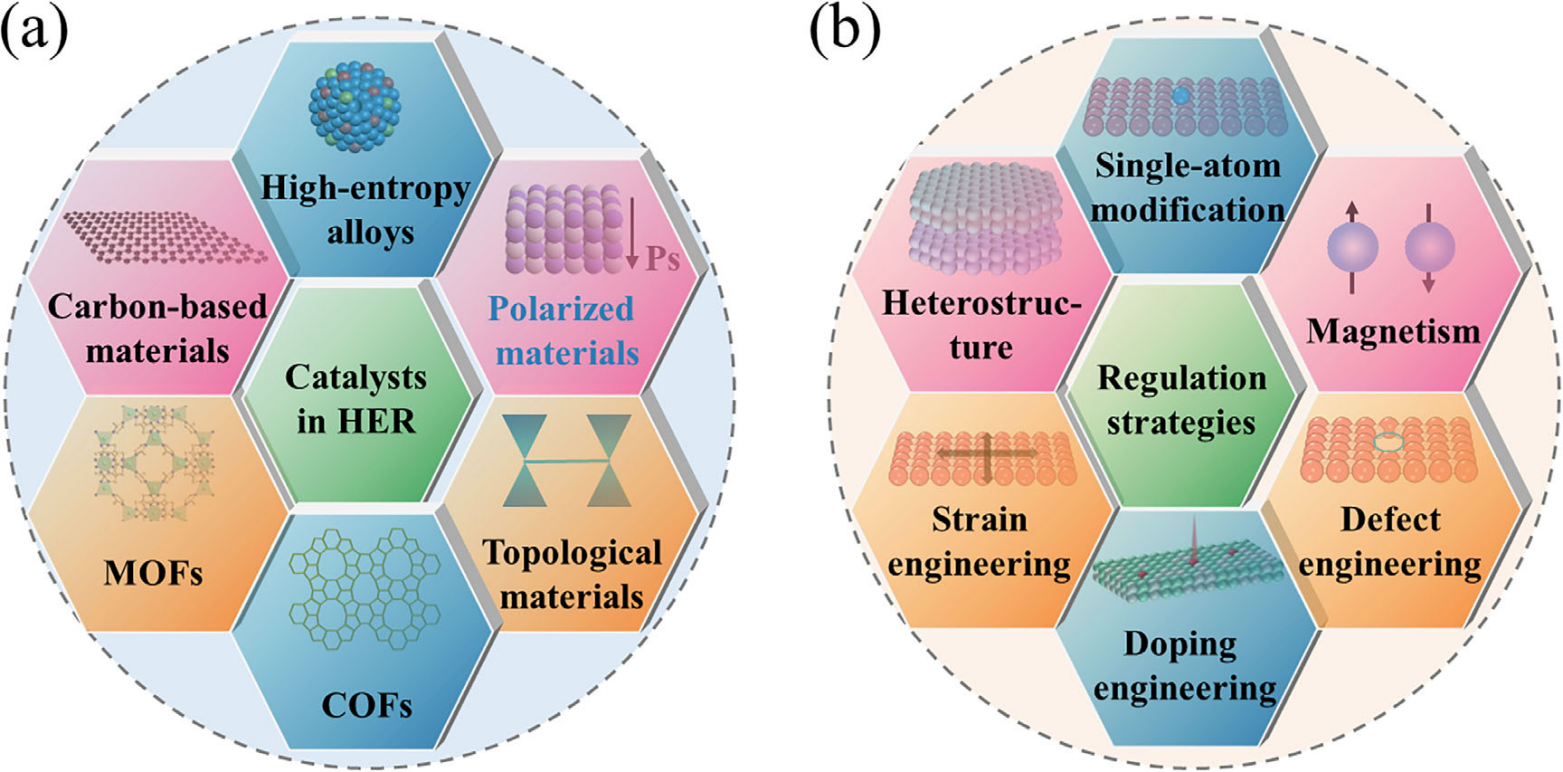
Current status of catalyst selection and regulation strategies for the HER. (a) Catalyst categories for the HER, including high‐entropy alloys, polarized materials, carbon‐based materials, and others (e.g., MOFs, COFs, and topological materials). This perspective focuses on ferroelectrics within the category of polarized materials. (b) Regulation strategies for enhancing catalytic activity: single‐atom modification, heterostructure construction, and other approaches (e.g., strain, doping, defect engineering, and magnetism) for the current catalysts.

To address these challenges, current research is primarily focused on three key strategies: (i) the development of non‐precious metal catalysts that retain high catalytic activity for efficient H_2_ production [6, 7, 8, 9]; (ii) the exploration of non‐water‐based H_2_ production pathways, such as catalytic decomposition of methane (CH_4_) and ammonia (NH_3_) [10, 11, 12]; and (iii) reducing reliance on electricity by utilizing semiconductor materials directly coupled with renewable energy sources—particularly solar energy—to drive photocatalytic hydrogen evolution [13, 14, 15, 16, 17, 18, 19]. Notably, all of these diverse hydrogen production pathways rely on the participation of various catalysts, making the development of novel catalysts [20, 21, 22, 23, 24, 25, 26, 27, 28] (Figure 1a)—such as high‐entropy alloys, polarized materials, and carbon‐based materials—and corresponding regulatory strategies (Figure 1b) [29, 30]—including single‐atom modification, heterogeneous structures, strain engineering, and others—a central focus in HER research.

**FIGURE 2:**
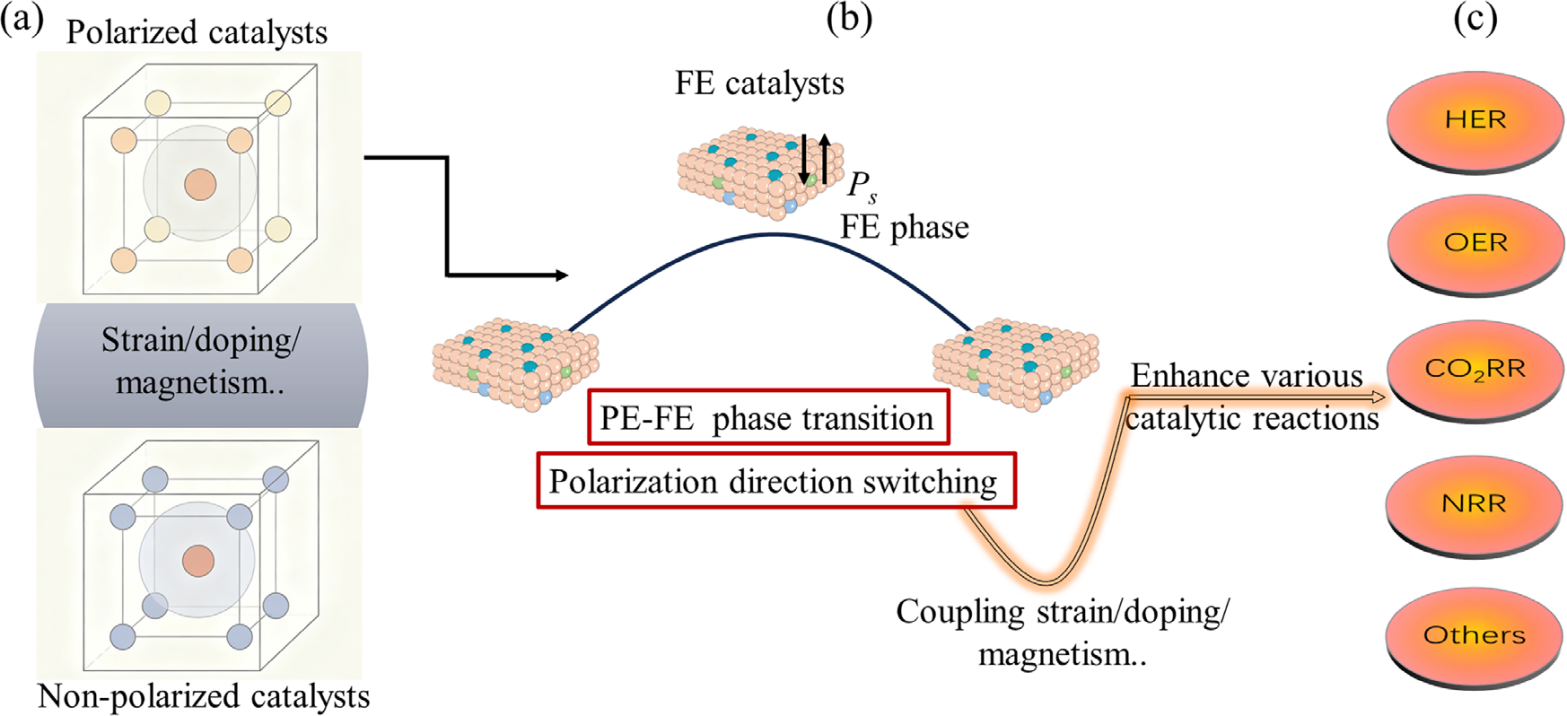
Coupling of intrinsic advantages of polarized materials and universal regulation strategies for applications in multiple catalytic reactions. (a) Schematic diagrams of polarized materials and non‐polarized materials, along with their common regulatory strategies, as depicted in Figure 1b. (b) The intrinsic mechanisms that can promote and control catalysis for FE materials (a subset of polarized materials), including polarization reversal and FE‐to‐PE phase transition, and can be leveraged in conjunction with other regulatory strategies to enhance various catalytic reactions, such as (c) HER, OER, CO_2_RR, and NRR.

Regarding the first category of alternative solutions—i.e., the development of non‐precious metal catalysts—extensive theoretical and experimental research has identified numerous candidate materials with the potential to substitute for traditional precious metals. These candidate materials can be broadly classified into the following four categories: (i) non‐precious metal compounds [6, 31, 32, 33, 34, 35, 36, 37, 38, 39, 40, 41, 42], (ii) intermetallic compounds [43, 44, 45], (iii) carbon‐based materials [25, 46, 47, 48, 49], and (iv) emerging advanced materials [50, 51, 52, 53, 54, 55, 56, 57, 58, 59, 60, 61, 62, 63]. Current research efforts are primarily focused on non‐precious metal compounds (Figure 2a), particularly transition metal phosphides [33, 34, 35] (e.g., Ni_2_P [35]), sulfides [36, 37] (e.g., MoS_2_ [37]), carbides [38, 39] (e.g., Mo_2_C [39]), and oxides [40, 41]. The core design strategies focus on the precise modulation of electronic structure, such as the *d*‐band center, and defect engineering—especially using non‐metallic vacancies—to optimize the intrinsic properties of active sites, thereby reducing the hydrogen adsorption free energy and effectively promoting the HER performance.

For intermetallic compounds, researchers leverage the synergistic effects among different non‐precious metals to modulate hydrogen adsorption energy barriers and facilitate water molecule dissociation, thereby enhancing their catalytic performance in alkaline media [43, 44, 45]. Additionally, carbon‐based materials, particularly graphene and carbon nanotubes, have attracted widespread attention due to their excellent conductivity, high specific surface area, and tunable structures [64]. Although intrinsic carbon materials exhibit low HER activity, the use of heteroatom doping (e.g., N, P, S) [46, 47, 48, 49] or loading carbon with single‐atom catalysts (e.g., forming M‐N‐C structures) [38, 65] can effectively construct highly active sites, leading to significant improvements in catalytic performance.

More recently, driven by research paradigms such as the Materials Genome Initiative, a series of emerging material systems has rapidly developed, including high‐entropy alloys [50], topological materials [23, 51, 52, 53, 54, 55], inorganic electrides [56, 57, 58, 59, 60], and metal–organic frameworks (MOFs) [61, 62]. These materials, with their abundant surface‐active sites, tunable electronic structures, topological surface states with high electron mobility, and unique geometric configurations, offer new platforms and boundless possibilities for advancing the understanding of catalytic mechanisms and achieving breakthroughs in HER performance.

The core of the second technological approach, i.e., exploring non‐water‐based H_2_ production pathways, involves circumventing water splitting by directly cracking hydrogen‐rich compounds to extract H_2_. Among these, CH_4_, NH_3_, and methanol (CH_3_COH) cracking have been the first to achieve large‐scale industrial application [10, 11, 12]. A key feature of this pathway is that H_2_ production is often coupled with the formation of valuable byproducts (compared to O_2_ produced in water splitting). However, compared to the relatively “pure” process of water splitting, such reactions tend to be more complex in nature. Moreover, most of these processes are not inherently carbon‐free.

For the third case, where the focus is on utilizing renewable energy to directly drive water splitting, photocatalytic technologies are a typical example [13, 14, 15, 16, 17, 18, 19]. Specifically, when a semiconductor material with an appropriate bandgap is photoexcited, electrons in the valence band can transition to the conduction band, forming electron–hole pairs. The excited electrons that move to the material's surface can then reduce H^+^ to H_2_. TiO_2_ is a representative material in the field of photocatalysis, but its wide bandgap and high charge recombination rate significantly limit its photocatalytic activity [66]. To address this issue, researchers commonly employ doping strategies [67], such as introducing metal (e.g., Fe/Cu/Mn) [68] or non‐metal elements (e.g., C and N) [69, 70], and using heterostructures (e.g., combining TiO_2_ with MoS_2_ or MOFs) [71, 72, 73], to reduce its bandgap and enhance solar photocatalytic performance. With the rapid development of high‐throughput screening and machine learning, a large number of ideal semiconductor materials for photocatalysis have been predicted and confirmed [14, 94, 95, 96, 97, 98, 99, 100, 101, 102, 103, 104], such as ferroelectric (FE) materials [74, 75, 76, 77, 78], perovskite materials [79, 80], and graphitic carbon nitride (g‑C_3_N_4_) [81], etc. Notably, this photocatalytic approach involves many candidate material systems from the first category capable of replacing precious‑metals. Therefore, these findings indicate that exploring semiconductor materials that are both based on non‑precious‑metal elements and have suitable bandgaps—enabling cost‐effective water splitting via photocatalytic pathways—represents a significant research direction.

### 1.2 Research Progress of FE Materials in H_2_ Production

In addition to classifying catalysts into metals and semiconductors based on their electronic structures, these materials can also be classified into non‐polarized and polarized catalysts according to the polarity of the materials (Figure 2a). For non‐polarized catalysts, engineering strategies such as strain regulation, doping, and surface passivation have been proposed to enhance their catalytic performance [77, 82]. Recently, ferroelectrics in polarized materials have gradually gained attention for their capability to regulate catalytic properties and drive distinct catalytic reactions through polarization direction switching or FE‐PE phase transitions [77, 83, 84, 85, 86] (Figure 2b). Remarkably, the regulatory strategies originally established for non‐polar catalysts are also applicable to polarized catalysts. However, the intrinsic properties of FE materials are absent in non‐polar materials. Moreover, this perspective focuses on FE materials, driven by two key considerations: First, previous studies have confirmed that numerous FE materials exhibit semiconducting or insulating characteristics. Second, the polarization properties of ferroelectrics offer a natural advantage for suppressing electron–hole recombination in photocatalytic processes. Together, these inherent properties provide promising opportunities for designing non‐precious‐metal‐based FE photocatalysts.

Based on previous research, spontaneous polarization influences surface reaction processes by modulating the adsorption strength of reactants on the catalyst surface, charge separation efficiency, and other key physicochemical properties [78, 87, 88, 89]. Analogous to the role of an external electric field in field‐effect transistors—which modulates interfacial electron transfer to affect gas–surface interactions [77, 90]—the spontaneous polarization in FE materials can also enhance reactant adsorption by regulating the chemical interactions and charge transfer between reactants and catalysts, because polarization can alter physicochemical characteristics, such as electronic structure and surface states [85, 89]. This adsorption enhancement effect is closely related to the polarization direction and arises from the changes in the direction and extent of electron transfer at the interface induced by different polarization orientations. Furthermore, variations in the polarization state not only regulate surface electron transfer but can also drive surface structural reconstruction of the material, manifested for example, as the reorientation of FE domains [83]. In heterogeneous catalytic reactions, the universal linear scaling relationships often exist between the adsorption energies of different intermediates on non‐polar catalyst surfaces, and this relationship constitutes a key bottleneck in independently optimizing the adsorption energies of each intermediate. In contrast, FE materials offer a pathway to overcome this limitation: through polarization‐induced surface reconstruction, a dynamically tunable catalytic surface can be achieved, thereby offering the potential to break the inherent structure‐activity relationship [77]. Consequently, the surface reaction processes in polarized catalysts are synergistically governed by domain orientation and surface polarization states, providing an effective strategy for the targeted optimization of electrocatalytic and photocatalytic performance.

In addition to significantly influencing the adsorption behavior of reactants, spontaneous polarization can also affect the electron–hole separation process in polarized catalysts [88]. In photocatalysis, polarization can facilitate the efficient separation of photogenerated carriers, thereby enhancing catalytic efficiency. This phenomenon has been observed in several systems, such as organic photovoltaics, AgBiP_2_Se_6,_ and BaTiO_3_, etc. [83, 88].

In electrocatalysis, polarization can modulate the chemical interactions and charge transfer between reactants and catalysts by altering physicochemical properties, such as electronic structure and surface states [85, 89]. For example, Ju et al. [83] found that the FE–paraelectric (PE) phase transition of AgBiP_2_Se_6_ can significantly modulate its photocatalytic activity and energy conversion efficiency. In the FE phase, the AgBiP_2_Se_6_ monolayer exhibits a higher photogenerated hole potential and enhanced driving force for water oxidation, whereas in the PE phase, photogenerated electrons possess a greater potential for the HER.

The effects of ferroelectric polarization can be used to enhance performance not only for water splitting reactions but also for a variety of other catalytic processes (Figure 2c), including the oxygen evolution reaction (OER), nitrogen reduction reaction (NRR), and CO_2_ reduction reaction (CO_2_RR) [83, 84, 85, 91, 92]. Furthermore, FE polarization exhibits significant potential in tuning catalytic selectivity, with its core mechanism lying in altering the surface's competitive adsorption capacity toward different reactants. For example, Guanwan et al. [91] employed ammonia oxidation as a model reaction and successfully achieved selectivity switching by modulating the polarization state of the BiFeO_3_/BiVO_4_ heterostructure: downward polarization enhances ammonia adsorption, favoring ammonia oxidation; upward polarization strengthens water adsorption, thereby driving the OER. Despite these broad applications of FE catalysts, the HER, due to its relatively simple reaction pathway, is often employed as a model system to facilitate a deeper understanding of the critical role of polarization in catalytic reactions.

In this perspective, we summarize three different processes for driving water splitting, covering thermocatalytic water splitting, electrocatalytic water splitting, and photocatalytic water splitting. Additionally, we present an overview of the principles and mechanisms of ferroelectricity. FE catalysts for the HER are then classified into the following categories: single‐phase FE catalysts (encompassing bulk and 2D systems), single‐atom modified FE catalysts, FE heterojunction catalysts, and other engineered FE catalysts (including strain‐induced piezoelectric effects and doping engineering). For each category, representative systems are discussed, and the catalytic mechanisms of FE catalysts for the HER are summarized from the perspectives of FE polarization, electronic structure, and surface vacancies, among others. Finally, we provide perspectives on the future development of FE catalysts for the HER with a focus on intrinsic FE metals, unconventional FE materials, and the coupling of spin with other physical properties.

## 2 Background

### 2.1 Processes for Catalytic Water Splitting

The current methods for water splitting mainly include three types: thermochemical, electrolytic, and photocatalytic. Unlike the latter two, thermochemical processes do not require a catalyst and directly split water into H_2_ and oxygen (O_2_) through high temperature (H_2_O → H_2_ + 1/2O_2_) [93]. The essential difference between electrolytic and photocatalytic water splitting lies in their driving mechanisms: the former relies on an external electrical current, while the latter is driven by light excitation. Both methods typically require the assistance of catalysts to facilitate the reaction.

**FIGURE 3:**
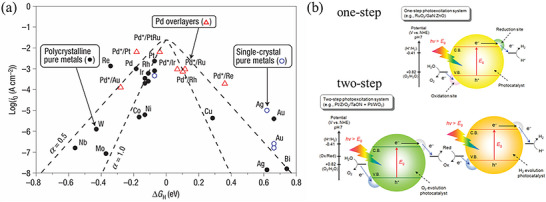
Theoretical criteria for evaluating the performance of electrocatalytic and photocatalytic water splitting. (a) Volcano plots of the HER for different pure metals with and without Pd metal overlayers [95]. Copyright 2006, Springer Nature. (b) Schematic energy level diagrams of one‐step and two‐step photocatalytic water splitting [96]. Copyright 2010, American Chemical Society.

In the electrocatalytic HER, which occurs on the cathode surface, two heterogeneous catalytic steps are involved: the initial Volmer reaction and the subsequent Heyrovsky or Tafel reaction. However, the actual kinetics of the HER are rather complex and influenced by the electrochemical potential [93]. Typically, the reaction proceeds via both the Heyrovsky and Tafel pathways simultaneously. An excellent electrocatalyst in experiments should meet several key criteria, including a low overpotential (e.g., at a current density of 10 mA cm^−2^), a small Tafel slope, and a high exchange current density (ideally within the range of 1–2 A cm^−2^) [94]. In theoretical calculations, the volcano plot is widely used to evaluate and compare the performance of different catalysts for the HER. In these plots, Pt is located at the top of the volcano because its hydrogen adsorption Gibbs energy change is close to zero (ΔG ≈ 0 eV), making it the best‐performing HER catalyst known to date [93, 95] (Figure 3a). Therefore, the development of low‐cost catalysts that are either entirely free of precious Pt or contain only minimal amounts of Pt (e.g., PtBi [95]), while still achieving catalytic activity comparable to that of pure Pt, would represent a significant step forward in HER research.

A key objective in photocatalytic water‐splitting research is the design of photocatalysts that operate efficiently within the visible‐light range while maintaining high performance and stability. Currently, photocatalytic water splitting strategies are mainly categorized into two types, as illustrated in Figure 3b.

The first approach utilizes a single visible‐light‐responsive photocatalyst to directly split water into H_2_ and O_2_. Such a catalyst must meet the following requirements for efficient solar‐to‐hydrogen conversion: appropriate band alignments to provide thermodynamic potential for water splitting, a sufficiently narrow bandgap to effectively absorb sunlight, and good stability against photodegradation [96]. Although the theoretical thermodynamic minimum bandgap for water splitting is 1.23 eV, practical photocatalytic systems generally require bandgaps exceeding 2.0 eV to compensate for overpotentials and kinetic barriers [14]. Due to these stringent conditions, only a limited number of photocatalysts are suitable for this strategy [97].

The second approach employs a two‐step excitation mechanism, in which two different photocatalysts are used to independently drive the H_2_ evolution and O_2_ evolution reactions. Compared to the one‐step system, the two‐step approach only requires that the H_2_ evolution catalyst meet the thermodynamic criterion for H_2_ evolution, while the O_2_ evolution catalyst must satisfy the corresponding criterion for O_2_ evolution. This relaxed constraint facilitates broader utilization of the visible‐light spectrum and enables efficient spatial separation of the H_2_ and O_2_ products [98]. However, this method still faces challenges, primarily in promoting electron transfer between the two semiconductors and suppressing reverse reactions involving redox mediators.

Moreover, to effectively utilize the visible and near‐infrared regions of the solar spectrum, the bandgap of the photocatalyst—regardless of whether it is employed in single‐step or two‐step configurations—must remain below 3 eV [14], ideally well below. As a result, precise engineering of the material's electronic band structure to enable absorption of low‐energy photons while preserving sufficient redox potential has become a critical focus in advancing this technology.

### 2.2 The Principles and Mechanisms of Ferroelectricity

Since the initial discovery of ferroelectricity in perovskite oxides, FE materials have remained a highly active and compelling research frontier. This enduring interest stems not only from the intriguing physical mechanisms underlying ferroelectricity, but also from the diverse functional properties these materials exhibit across a wide range of applications.

**FIGURE 4:**
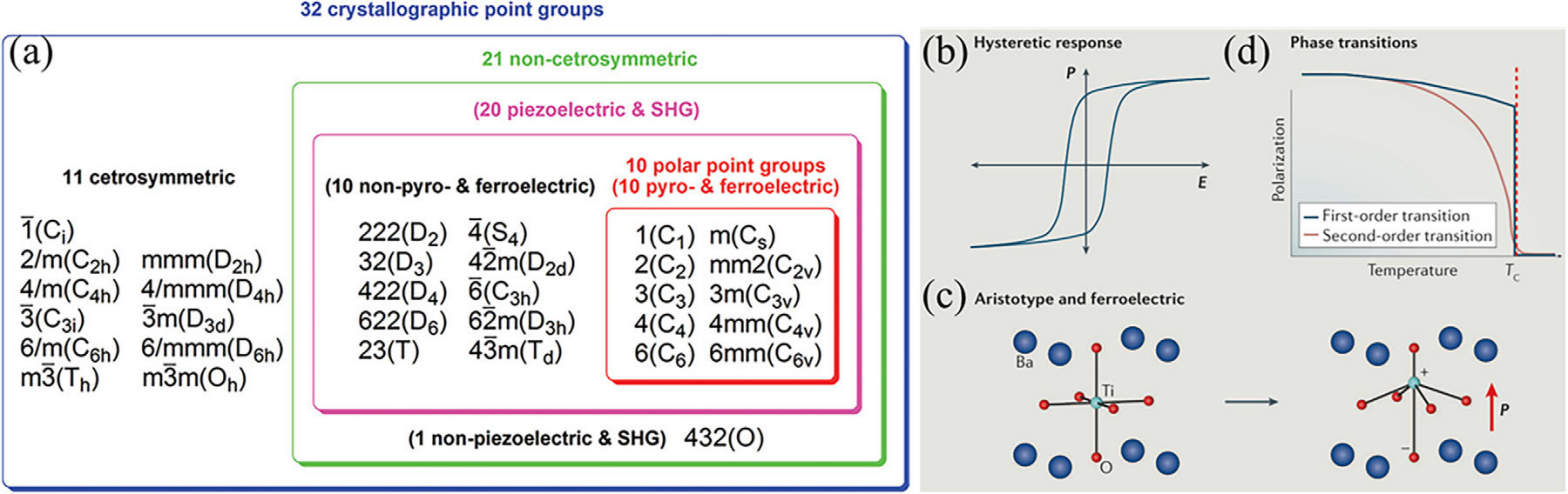
Point groups and evaluation criteria of traditional FE materials. (a) The relationship between ferroelectricity, thermoelectricity, piezoelectricity, and second harmonic generation (SHG) based on symmetry analysis [99]. Copyright 2012, American Chemical Society. (b) The hysteretic response of FE materials. (c) Crystal structures of the aristotype (high‐symmetry phase) and ferroelectric phase of BaTiO_3_. (d) The first‐order and second‐order transitions of FE materials [100]. Copyright 2017, Springer Nature.

FE materials constitute a special class of dielectrics, forming a subcategory of pyroelectric and piezoelectric materials. The key feature that distinguishes them from non‐FE materials is their spontaneous polarization; the direction of polarization can be reversed by applying an external electric field [99]. By definition, the space group of a FE material must belong to one of the ten polar point groups (PGs), namely *C_1_*, *C_s_*, *C_2_*, *C_2v_*, *C_3_*, *C_3v_*, *C_4_*, *C_4v_*, *C_6_*, and *C_6v_* (Figure 4a). This implies that only materials with these PGs can potentially exhibit ferroelectricity.

For a FE phase, the polarization (P) versus electric field (E) (P–E) curve displays a characteristic hysteresis loop (Figure 4b), which serves as a direct macroscopic manifestation of ferroelectricity. It reflects the switching of FE domains or polarization directions below the dielectric breakdown field. Typically, ferroelectricity occurs only below the Curie temperature (*T*_c_). Above *T*_c_, the material transitions into its aristotype (high‐symmetry phase) (Figure 4c). Additionally, these FE phase transitions can be classified into displacement‐type (Type‐I) and order‐disorder‐type (Type‐II) based on their microscopic mechanisms (Figure 4d). For instance, BaTiO_3_ is a type‐I FE material, where spontaneous polarization arises from the relative displacement of positive and negative ions in the crystal lattice [100]. In contrast, NaNO_2_ is a typical order‐disorder‐type FE material, whose ferroelectricity mainly originates from the ordered alignment of $NO_{2}^{-}$ ion orientations [101].

**FIGURE 5:**
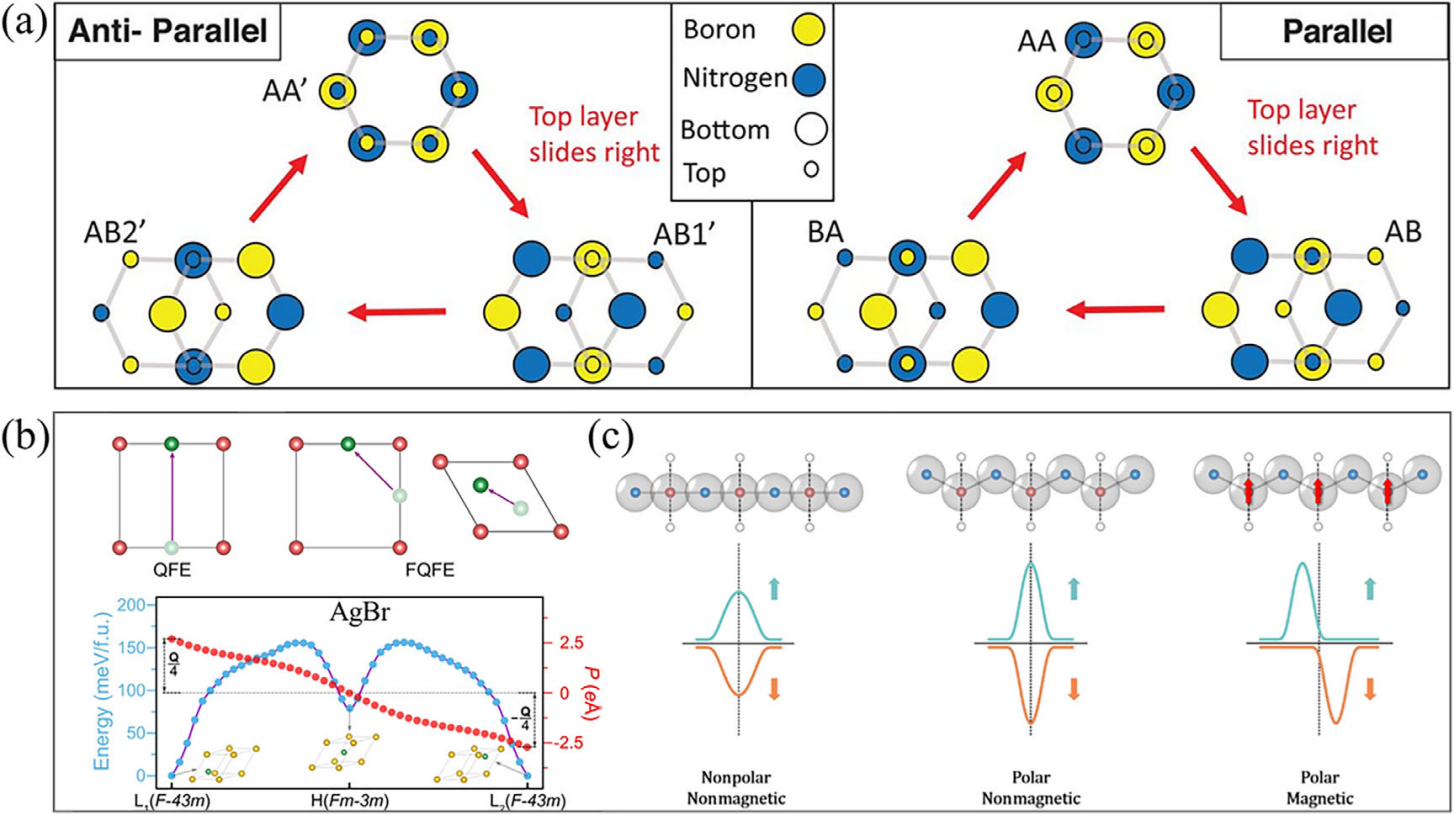
Mechanisms of emerging FE materials (sliding ferroelectrics/quantum fractional ferroelectrics/type‐III multiferroics). (a) Schematic diagram of the FE polarization generated by parallel and antiparallel slip in double‐layer stacking structures [102]. Copyright 2021, American Association for the Advancement of Science. (b) Top panel: Schematic diagrams of quantum ferroelectricity (QFE) and fractional quantum ferroelectricity (FQFE). Bottom panel: The energy barrier associated with the transformation of AgBr from the $F\bar{4}3m$ phase to the Fm‐3m phase was calculated using the nudged elastic band (NEB) method. The calculation considered the variation in polarization intensity along the reaction path, which involved moving a Br atom from position (1/4, 1/4, 1/4) to (1/2, 1/2, 1/2) and finally to (3/4, 3/4, 3/4) within a primitive cell [105]. Copyright 2024, Springer Nature. (c) Schematic diagrams of FE‐driven magnetic behavior in a 1D ionic chain, along with the corresponding density of states (DOS) diagrams [107]. Copyright 2025, American Physical Society.

More recently, research in the field of FE materials has broken through traditional frameworks, achieving significant progress at the mechanistic level. A range of novel FE mechanisms beyond the classic ionic displacement models have been discovered and have become frontier hotspots (Figure 5a–c), represented by sliding ferroelectricity (e.g., ReSe_2_) [102, 103, 104], fractional quantum ferroelectricity (e.g., AgBr) [105, 106], and type‐III multiferroicity (coupling of ferroelectricity and magnetism) [107, 108, 109]. The emergence of these mechanisms is expanding the conceptual scope of FE physics into new and broader dimensions.

Several newly developed FE materials have been successfully synthesized experimentally; however, their application in the HER is still in its early stages. Taking type‐III multiferroics as an example, we propose a practical experimental strategy. Leveraging the additional magnetic degree of freedom, type‐III multiferroics allow independent control of magnetic and FE transition temperatures. Comparative catalytic activity measurements across polarized and non‐polarized, as well as magnetic, non‐magnetic, and magnetoelectric‐coupled states, will help elucidate the distinct roles of FE polarization and magnetism in governing the catalytic process.

### 2.3 Mechanism of FE Catalysis in HER

**FIGURE 6:**
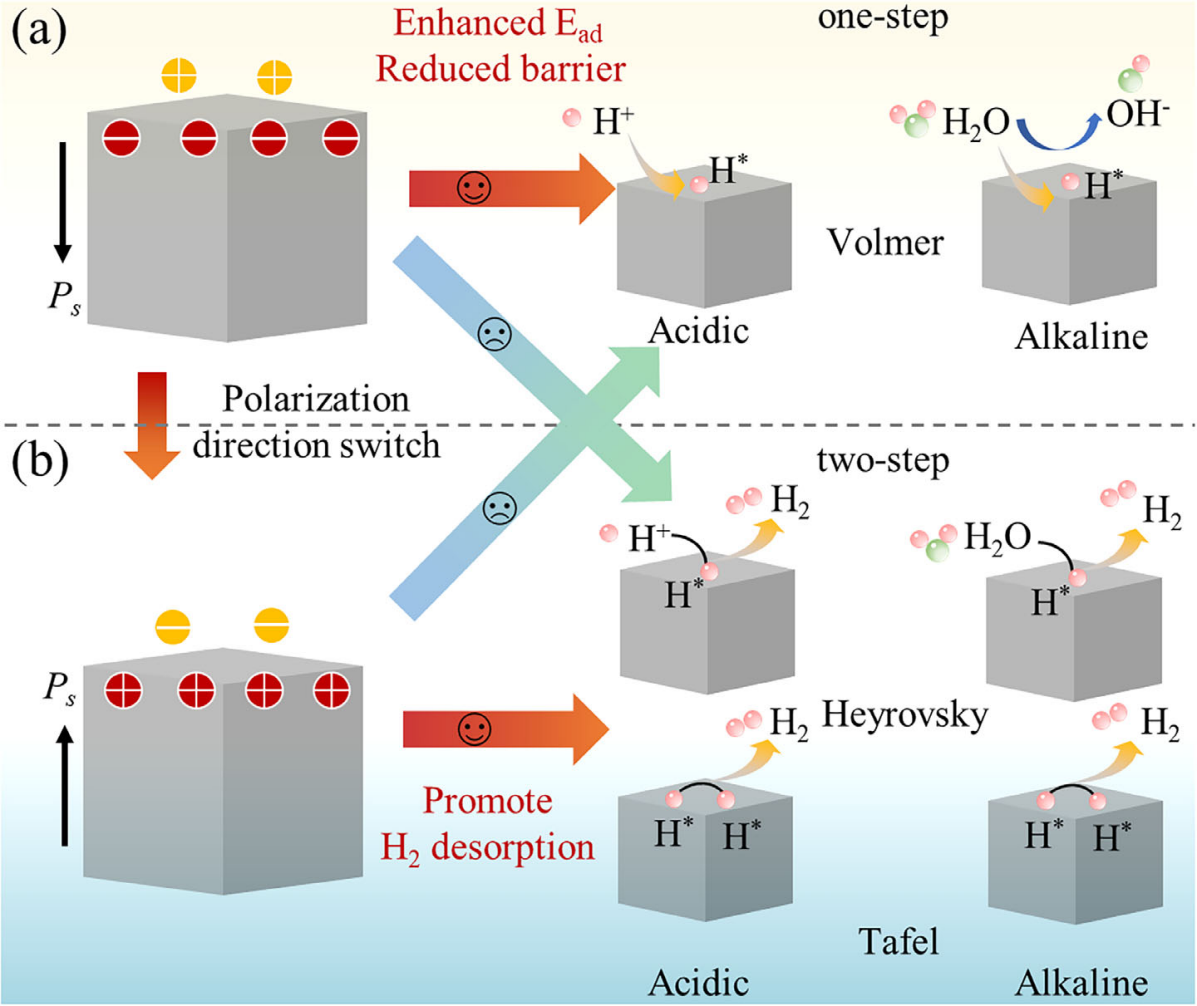
Ferroelectric polarization modulation of HER reaction pathways in acidic and alkaline electrolytes. (a) Downward polarization (*P*_s_↓) enhances H^*^ adsorption and water dissociation to accelerate the Volmer step. (b) Upward polarization (*P*_s_↑) weakens H^*^ adsorption to promote the Heyrovsky/Tafel steps for efficient H_2_ generation.

The reversal of the FE polarization direction systematically modulates the surface charge distribution of the catalyst and the binding energy of adsorbed hydrogen intermediates (H^*^), thereby tuning the kinetics of each elementary step of the HER under both acidic and alkaline conditions. Consequently, it influences whether the reaction proceeds via the Volmer–Heyrovsky or Volmer–Tafel pathway. Specifically, when the FE polarization direction points downward (*P*_s_↓), the surface becomes electron‐enriched. Under acidic conditions, this enhances H^+^ adsorption; under alkaline conditions, these surface electrons significantly lower the dissociation energy barrier of H_2_O (Figure 6a). However, excessively strong adsorption can suppress the subsequent Heyrovsky and Tafel desorption processes. In contrast, when the polarization direction points upward (*P*_s_↑), the FE material surface is enriched with holes. Under acidic conditions, this promotes the recombination of H^*^ with either H^+^ in the solution (Heyrovsky step) or adjacent H^*^ (Tafel step); under alkaline conditions, it simultaneously optimizes water dissociation and H desorption, thereby overcoming the kinetic limitations of alkaline HER and shifting the reaction pathway toward a synergistic Volmer‐Heyrovsky and Volmer‐Tafel mechanism [78] (Figure 6b).

## 3 Representative FE Materials for Catalysis of the HER

FE materials exhibit unique potential for catalysis of the HER due to their intrinsic spontaneous polarization. This polarization effect can effectively modulate the surface charge distribution of catalysts, optimize the adsorption energy of hydrogen intermediates, and thereby enhance the intrinsic activity for the reaction. Based on this, this perspective will summarize the research progress in this field from the following four directions: single‐phase FE catalysts (including bulk/2D FE catalysts), FE single‐atom modified catalysts, FE heterostructure catalysts, and other engineered FE catalysts (including using strain and doping).

### 3.1 Single‐Phase FE Catalysts

#### 3.1.1 Bulk Example: Perovskite BaTiO_3_

**FIGURE 7:**
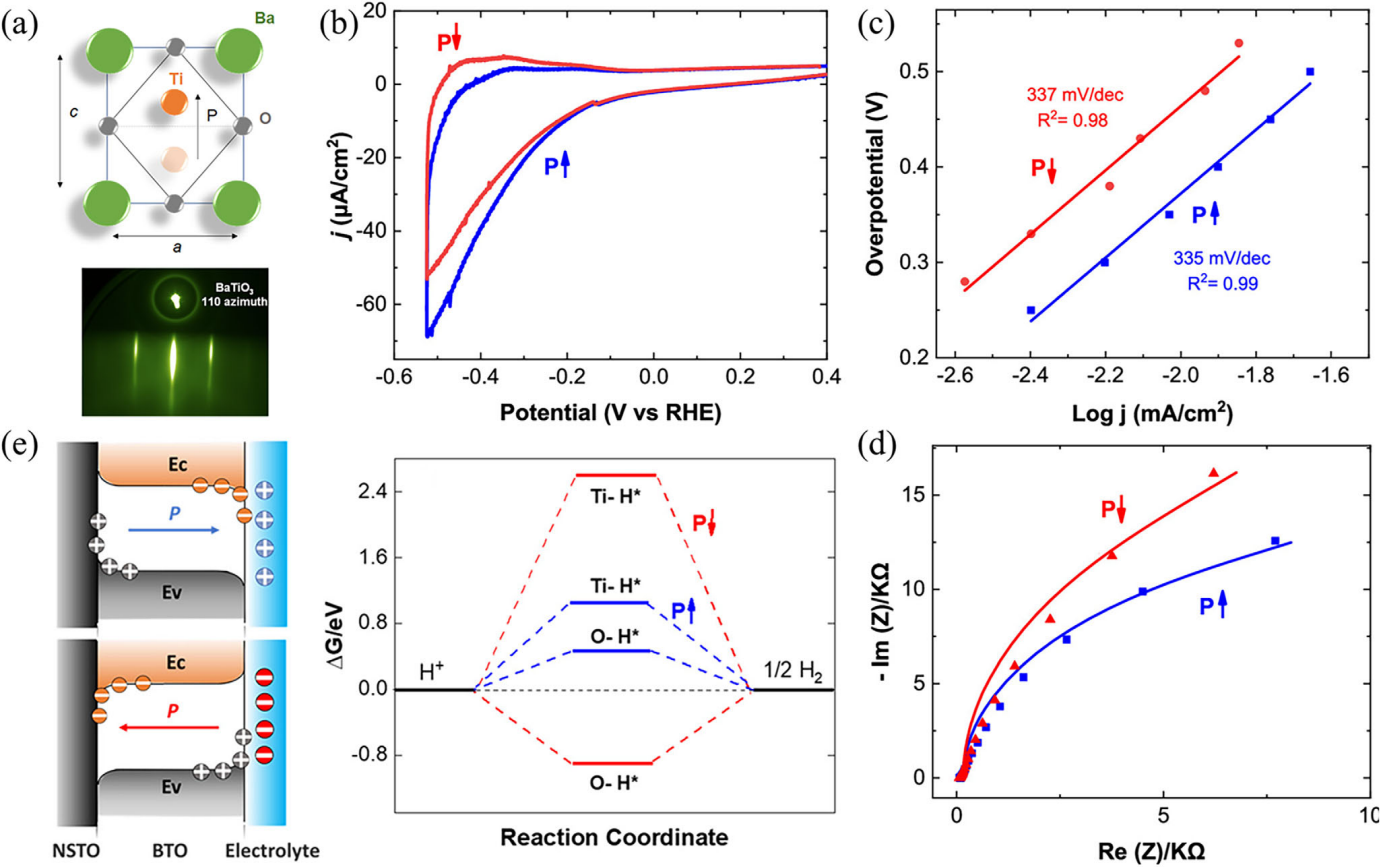
HER performance of bulk FE catalyst BaTiO_3_. (a) Top panel: The crystal structure of BaTiO_3,_ in which the Ti atom moves from the downward polarized position to the upward polarized position in the O‐coordinated octahedron. Bottom panel: The reflection high‐energy electron diffraction pattern of BaTiO_3_ thin films observed along the [108] direction. The relationship between the HER performance and the FE polarization, where upward polarization and downward polarization are respectively shown in blue and red: (b) cyclic voltammetry, (c) Tafel slopes, and (d) electrochemical impedance spectra. (e) Schematic illustration of the effect of FE polarization in BaTiO_3_ on band bending, along with the theoretically calculated Gibbs energy changes during the HER steps for the two polarization directions [110]. Copyright 2022, American Chemical Society.

As a lead‐free and chemically stable FE material, BaTiO_3_ has emerged as a key model system for investigating the influence of polarization effects on the HER. Recent studies have revealed that FE polarization can directly regulate the HER activity on BaTiO_3_ surfaces [80, 88, 110]. For instance, Abbasi et al. [110] experimentally demonstrated that switching the polarization direction of a BaTiO_3_ thin film from upward to downward leads to two distinct electrocatalytic behaviors on the same catalyst surface, as shown in Figure 7.

In this work, a BaTiO_3_ single crystal film with (001) orientation was prepared by an epitaxial growth method, as shown in Figure 7a. Cyclic voltammetry (Figure 7b) and Tafel slope analysis (Figure 7c) reveal that the upward‐polarized BaTiO_3_ surface exhibits a more negative onset potential and a higher exchange current density compared to the downward‐polarized surface, confirming significantly enhanced HER kinetics under upward polarization. Furthermore, electrochemical impedance spectroscopy (Figure 7d) showed that the upward‐polarized BaTiO_3_ film displayed a markedly smaller semicircle radius, indicating reduced charge transfer resistance and accelerated interfacial electron transfer. DFT+U calculations revealed the polarization modulation mechanism on the BaTiO_3_‐(001) surface: upward polarization reduces the HER barrier by lowering the work function (Figure 7e). Overall, this study provides direct evidence for the reversible modulation of the HER process by FE polarization switching, achieving precise “on‐demand” control of catalytic activity through polarization direction.

Furthermore, phenomena similar to those observed for BaTiO_3_ during the HER have also been reported for certain other bulk single‐phase FE catalysts, including both perovskite and non‐perovskite systems such as Bi_3_TiNbO_9_, Bi_2_Fe_4_O_9_, etc. [80, 111, 112, 113]—although the majority adopt a perovskite structure.

#### 3.1.2 2D Examples: CuInP_2_S_6_ and AgBiP_2_Se_6_

**FIGURE 8:**
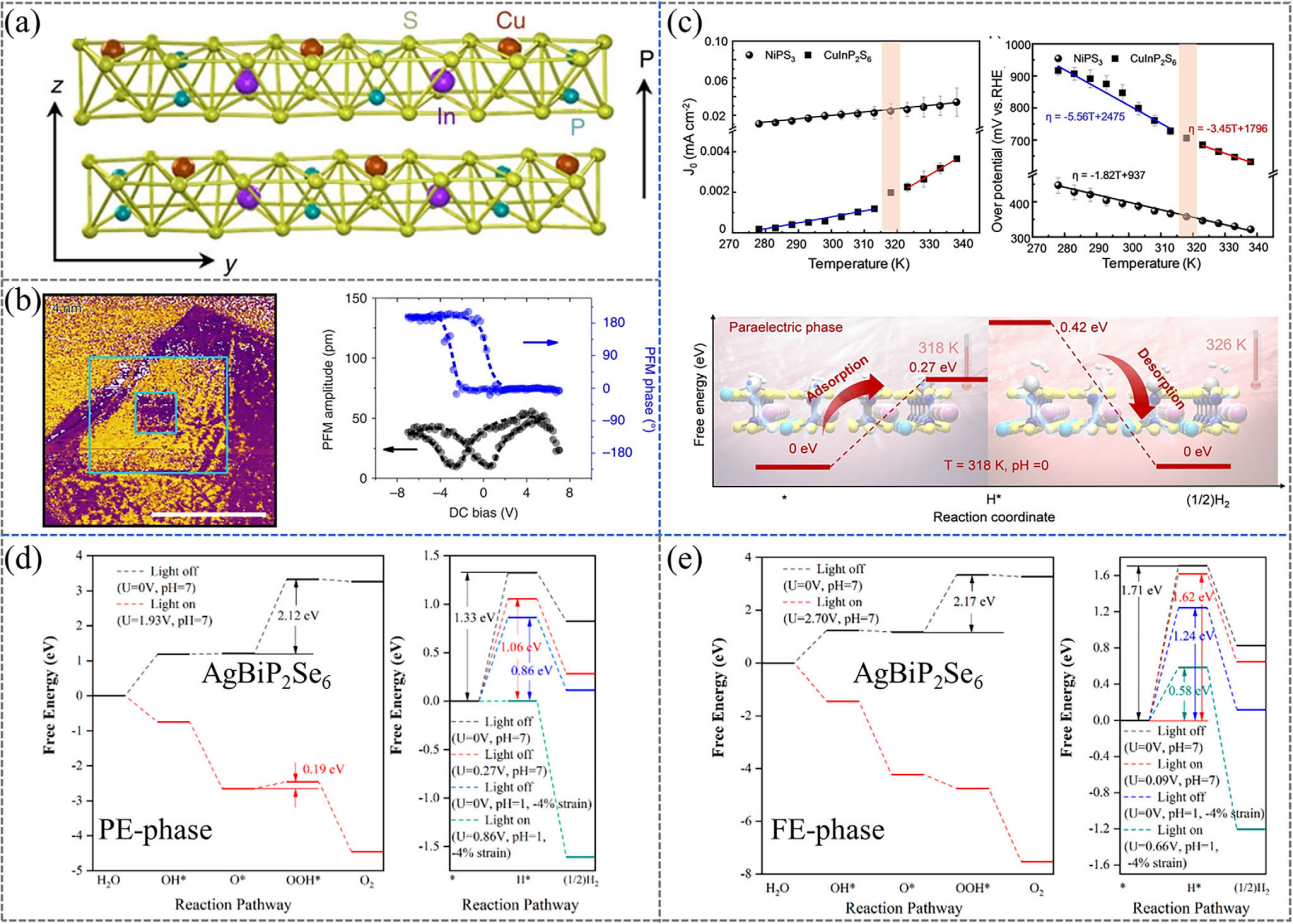
HER performance of the 2D FE catalyst XYP_2_Z_6_ (X = Cu, Ag; Y = Bi, P; Z = S, Se). (a) The crystal structure of CuInP_2_S_6_. (b) Piezoresponse force microscopy image of a CuInP_2_S_6_ film with a thickness of 4 nm, along with the corresponding amplitude (black) and phase (blue) hysteresis loops [114]. Copyright 2016, Springer Nature. (c) Top panel: Exchange current density and overpotential of CuInP_2_S_6_ as a function of temperature (270 K – 340 K). Bottom panel: The Gibbs (free) energy of the HER on CuInP_2_S_6_ in the paraelectric phase near the FE‐PE phase transition temperature (T_C_ = 318 K) [75]. Copyright 2024, Wiley‐VCH GmbH. Gibbs (free) energy profiles for the OER (left panel) and HER (right panel) on both the (d) PE‐phase and (e) PE‐phase of a AgBiP_2_Se_6_ monolayer under both light and dark conditions, considering the effects of applied potential (U), pH, and a ‐4% biaxial strain [83]. Copyright 2019, American Chemical Society.

Compared with bulk materials, 2D materials offer significant advantages in regulating physical and chemical properties by reducing dimensions. This section will take the single‐phase 2D catalysts CuInP_2_S_6_ and AgBiP_2_Se_6_ as typical examples to summarize the influence of polarization and FE‐PE phase transitions on both electrocatalytic and photocatalytic H_2_ production, as shown in Figure 8.

Liu et al. [114] reported that 2D CuInP_2_S_6_ exhibits surface ferroelectricity at room temperature, with a phase transition to a PE phase at approximately 320 K. Figure 8a shows the crystal structure of 2D CuInP_2_S_6_. For films with a thickness of ∼4 nm, switchable polarization was observed (Figure 8b). Furthermore, not only does the 2D FE material CuInP_2_S_6_ display out‐of‐plane vertical polarization, but similar polarization orientation has also been observed for a α‐In_2_Se_3_ monolayer [115]. These findings have stimulated extensive exploration into layered FE materials. In particular, building upon the CuInP_2_S_6_ system, researchers have discovered a series of ABP_2_X_6_ (A = Cu, Ag; B = In, Bi, Cr, V; X = S, Se)‐structured materials that also exhibit ferroelectricity [116], some of which additionally possess magnetism (e.g., CuVP_2_Se_6_), making them ideal candidates for investigating multiferroic coupling.

Typically, due to the semiconducting behavior of CuInP_2_S_6_, the study of its surface chemical properties often relies on photoexcitation techniques. However, Wang et al. [75] achieved reversible switching between the FE and PE phases of CuInP_2_S_6_ through temperature control and further investigated its electrocatalytic HER. They compared CuInP_2_S_6_ with the non‐polar catalyst NiPS_3_ and observed significantly different electrocatalytic behaviors within the temperature range of 313–318 K. Specifically, NiPS_3_ showed a strong linear dependence between overpotential and exchange current density, while CuInP_2_S_6_ exhibited a sudden discontinuity. This anomaly, observed precisely within its FE‐PE phase transition range of 313–318 K, was attributed to the FE transition, as it deviates completely from the linear trend that governs its behavior outside this temperature window (Figure 8c). Moreover, their study found that an increase in temperature provides an additional driving force to overcome the reaction activation energy, thereby significantly enhancing the catalytic activity, as evidenced by the gradual increase in exchange current density and the decreasing trend in overpotential. Meanwhile, they pointed out that due to the semiconducting nature of CuInP_2_S_6_ and its limited exposed active sites, its catalytic performance is weaker than that of NiPS_3_. The study suggests that the variation in the electrocatalytic HER behavior of CuInP_2_S_6_ under thermal stimulation is mainly governed by its intrinsic FE‐PE phase transition. Although CuInP_2_S_6_ exhibits unsatisfactory performance in electrocatalysis, Xu et al. [117] discovered that it shows great promise in photocatalysis. Their study reveals that CuInP_2_S_6_ has a bandgap of approximately 2.31 eV, endowing it with strong visible‐light absorption capability. More importantly, its characteristic out‐of‐plane polarization effectively facilitates the separation of photogenerated electrons and holes, positioning it as a highly promising photocatalyst for visible‐light‐driven water splitting.

Based on the study by Wang et al. [75], it is evident that the T_C_ significantly affects the HER of FE catalysts. It is well known that FE catalysts exhibit a FE phase below the *T*_C_ and a PE phase above it. Therefore, when investigating the effect of polarity on the HER, the reaction conditions should generally be maintained below the T_C_. If the T_C_ is above room temperature, the reaction can be carried out under open‐air conditions. In addition, above the T_C_, the catalyst transitions to the PE phase, which may lead to two scenarios: first, the catalytic activity decreases; second, the catalyst retains relatively high activity under thermal excitation [75]. However, the influence of the reaction temperature vs. T_C_ is often neglected in some studies on the application of FE catalysts to the HER.

Ju et al. [83] systematically explored the influence of FE switching on the photocatalytic water splitting behavior (including both HER and OER) of 2D AgBiP_2_Se_6_, comparing its FE and PE phases from a theoretical perspective, as illustrated in Figure 8d,e. Their study revealed that there are differences between the FE and PE phases of 2D AgBiP_2_Se_6_ in terms of the exciton binding energy, redox capacity, and the driving force of photogenerated carriers. These differences enable the 2D AgBiP_2_Se_6_ to facilitate the HER and OER in the FE and PE phases, respectively. Specifically, the FE phase exhibits a stronger driving force for the transfer of photogenerated holes, thereby favoring the OER during water splitting. In contrast, the PE phase shows a more pronounced driving force for the transfer of photogenerated electrons, which enhances its reductive capability and allows it to promote the HER. Furthermore, through calculating the reaction pathways for OER and HER in each phase, the authors demonstrated that the FE phase serves as an ideal photocatalyst for OER, while the PE phase is more favorable for enhancing HER activity under acidic conditions and under biaxial compressive strain (−4%). Although this study primarily focused on exploring the application potential of FE and PE phases for different catalytic reactions (e.g., HER and OER), they also pointed out that in the FE phase of AgBiP_2_Se_6_, the built‐in electric field induced by polarization significantly suppresses the recombination of electrons and holes. This results in a much higher carrier utilization efficiency in the FE phase compared to the PE phase. Consequently, they found that the FE phase exhibits significantly better solar energy utilization efficiency during HER and OER processes than the PE phase.

In addition to the aforementioned studies, several investigations have explored the HER performance of other single‐phase 2D FE catalysts, such as In_2_Se_3_, GeS, and Hf_2_Ge_2_S_6_ [92, 118, 119]. Overall, these studies consistently demonstrate that the distinct exciton binding energies and driving forces for transfer of photogenerated carriers in the FE and PE phases lead to their markedly different catalytic performance for the OER and HER in water splitting. Moreover, in the FE phase, the intrinsic polarization significantly suppresses electron–hole recombination, thereby enhancing the availability of photogenerated charge carriers for water splitting.

### 3.2 FE Single‐Atom Modified and Heterostructure Catalysts

In this perspective, single‐atom modified FE catalysts and FE heterostructure catalysts are discussed together because both are based on FE materials as functional substrates [120, 121]. Research on these FE catalyst structures leverages either the polarization properties of the substrate or charge transfer across interfaces to modulate and enhance the catalytic performance of additional catalyst layers or single atoms for the HER. Specifically, when single atoms are anchored (modified) on FE materials, the FE polarization can directly modulate the catalytic behavior of the active sites. In contrast, in heterostructure FE catalysts (involving layer‐layer coupling), polarization switching tends to drive charge accumulation or depletion at the interfaces, thereby indirectly influencing the surface catalytic activity. Nevertheless, in real catalytic systems, these two mechanisms often coexist and interweave, making them difficult to separate.

Furthermore, for single‐atom (typically transition metals (TMs)) modified FE catalysts used in water splitting, numerous studies have demonstrated that the polarization direction can regulate the surface charge distribution, thereby enabling the modulation of the transition metal *d*‐band center and ultimately affecting its catalytic activity [122]. Specifically, when the FE polarization direction points downward, electrons are typically transferred from the FE lattice to the anchored TM sites. This transfer increases the *d*‐orbital occupancy and enhances intra‐*d*‐shell electron–electron repulsion. Furthermore, interfacial lattice distortion and TM‐substrate orbital hybridization become stronger, collectively driving the *d*‐band center upward toward the Fermi level [120].

In heterostructure or single‐atom FE catalysts, it is often difficult to determine whether performance enhancement arises from direct polarization modulation of active sites or from indirect charge transfer across interfaces. Based on our review and understanding of the relevant work, when single atoms are anchored (modified) on FE materials, the FE polarization can directly modulate the catalytic behavior of the active sites. In contrast, in heterostructure FE catalysts (involving layer–layer coupling), polarization switching tends to drive charge accumulation or depletion at the interfaces, thereby indirectly influencing the surface catalytic activity. Nevertheless, in real catalytic systems, these two mechanisms often coexist and interweave, making them difficult to separate.

#### 3.2.1 Single‐Atom Modified FE Catalysts: TM Atoms‐CuInP_2_S_6_

**FIGURE 9:**
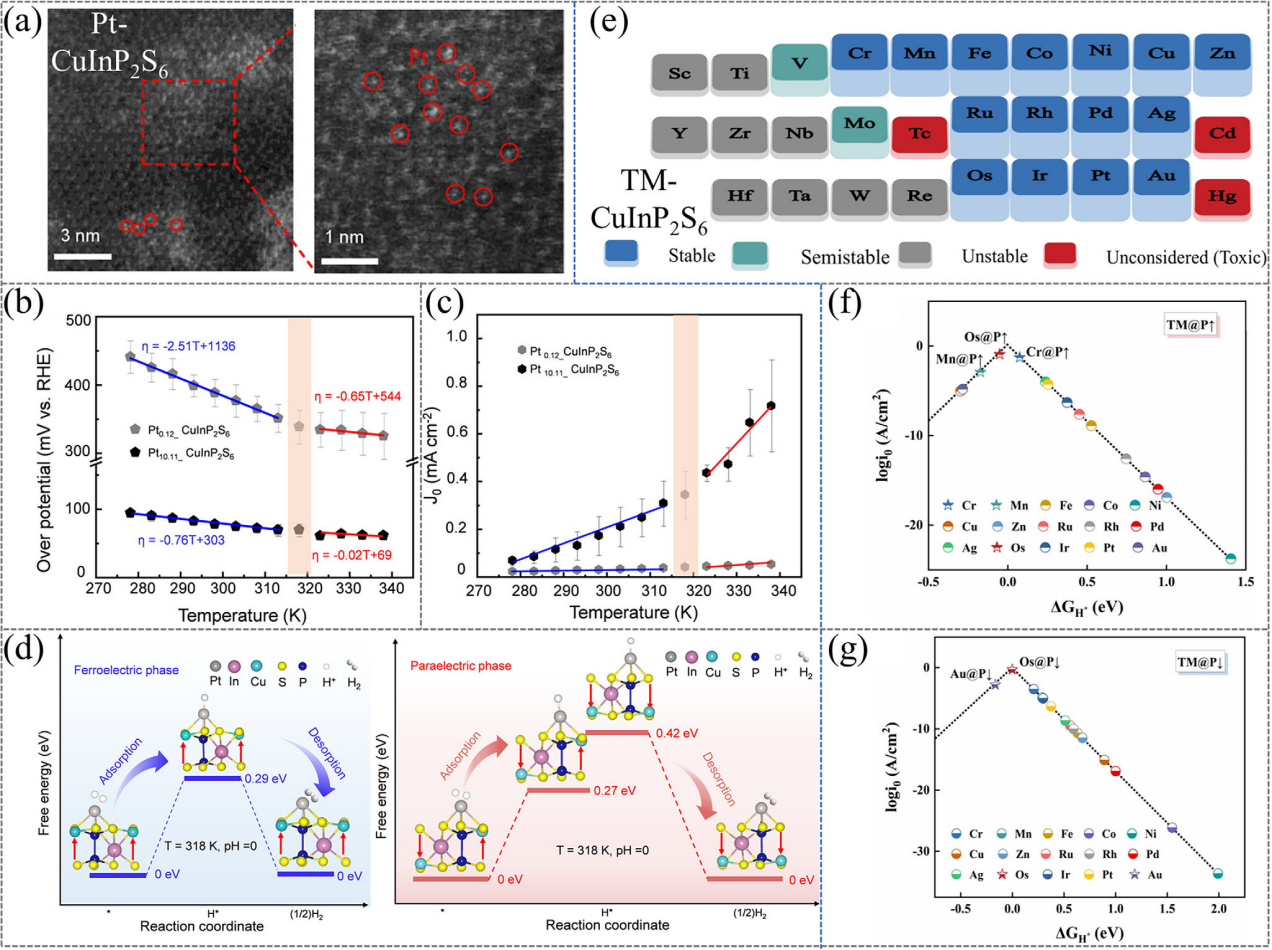
HER performance of single‐atom modified FE catalysts. (a) High angle annular dark field‐scanning transmission electron microscope (HAADF‐STEM) images of Pt‐CuInP_2_S_6_. (b) Exchange current density and (c) overpotential of Pt_0.12/10.11_/CuInP_2_S_6_ as a function of temperature (270–340 K). (d) The Gibbs (free) energy profiles for the electrocatalytic HER on both FE and PE phases Pt‐CuInP_2_S_6_ nanosheets at 0 V [75]. Copyright 2024, Wiley‐VCH GmbH. (e) Stability screening of TM atoms anchored on 2D CuInP_2_S_6_. (f, g) Gibbs energy diagrams of the HER for selected TM atoms anchored on 2D CuInP_2_S_6_ with upward and downward FE polarization, respectively [123]. Copyright 2026, Elsevier BV.

To reduce reliance on precious metals and to enhance the activity of various catalysts, researchers have utilized the *d*‐band center theory through anchoring noble transition metal atoms onto substrate surfaces, thereby improving overall catalytic performance [6, 122]. In this context, FE materials also show promise as potential substrates. By loading single atoms or nanoparticles on their surfaces and leveraging the tunability of FE polarization, the electronic structure of the single‐atom active centers can be precisely regulated, further boosting the intrinsic catalytic activity. Using TM‐CuInP_2_S_6_ as a representative model system [75, 123], this section will summarize the mechanistic roles of both FE‐PE phase transitions and FE polarization switching in modulating the HER on TM‐loaded CuInP_2_S_6_, as shown in Figure 9.

As discussed in Section 3.1.2, Wang et al. [75] investigated the HER performance of the 2D FE material CuInP_2_S_6_ as a single‐phase catalyst. Due to its intrinsic semiconductor characteristics, the performance of the single‐phase FE catalyst CuInP_2_S_6_ in electrocatalytic water splitting is unsatisfactory. Subsequently, they introduced Pt single atoms onto the surface of CuInP_2_S_6_ by electrochemical deposition and confirmed the successful loading of Pt using transmission electron microscopy and X‐ray absorption spectroscopy (Figure 9a). By controlling the deposition time, they further regulated the loading amount of Pt on the CuInP_2_S_6_ surface. Performance tests revealed that, compared to pure CuInP_2_S_6_ (Figure 8c), the Pt*_x_*‐CuInP_2_S_6_ catalyst exhibited a significantly reduced overpotential and an enhanced exchange current density for the HER (Figure 9b,c), indicating a remarkable improvement in its HER performance. Notably, both Pt*_x_*‐CuInP_2_S_6_ and CuInP_2_S_6_ exhibited similar temperature‐dependent behavior: near the *T*_C_, both overpotential and exchange current density showed distinct discontinuities, which were attributed to the phase transition from FE to PE. Furthermore, they emphasized that, for CuInP_2_S_6_, the origin of FE polarization lies in the out‐of‐plane displacement of Cu atoms relative to the S atomic planes. Consequently, the polarization state modulates H^+^ adsorption and desorption kinetics by governing the positional occupancy of Cu atoms. In the FE phase, Cu atoms are located within the S atomic planes, allowing the adsorption of H^+^ intermediates, but the desorption of H_2_ is relatively difficult. In contrast, in the PE phase, thermally activated Cu hopping between the upper and lower S planes enhances both H^+^ adsorption and H_2_ desorption—thereby improving overall HER kinetics (Figure 9d). This work reveals that, in the context of electrocatalytic water splitting, FE single‐atom catalysts can effectively regulate the catalytic activity of the HER by leveraging the atomic hopping mechanism induced by polarization transition.

Unlike the previous work, Wang et al. [123] employed theoretical calculations to investigate the regulatory mechanism of polarization direction on the activity of single‐atom modified FE catalysts. After excluding unstable or toxic TM atoms, they screened 15 stable TM atoms (including Cr, Mn, Fe, Co, Ni, Cu, Zn, Ru, Rh, Pd, Ag, Os, Ir, Pt, and Au) for use in a TM‐CuInP_2_S_6_ catalyst system (Figure 9e). Their results indicate that changing the polarization direction significantly affects the adsorption free energy of H intermediates, thereby modulating the HER overpotential. Taking Cr@P↑‐CuInP_2_S_6_ and Mn@P↑‐CuInP_2_S_6_ as examples, they exhibit low overpotentials in the upward polarization state (0.07 and 0.17 eV, respectively), whereas the overpotentials increase to 0.21 and 0.29 eV in the downward polarization state (Figure 9f,g). Furthermore, they pointed out that the change in polarization direction causes a reversal of the built‐in electric field within the CuInP_2_S_6_ substrate, which in turn regulates the local electron density and *d*‐band center position of the TM atoms. Upward polarization promotes electron accumulation at the TM sites, raising the *d*‐band center and enhancing the adsorption capacity for H intermediates; conversely, downward polarization weakens the adsorption strength—optimizing the overall HER pathway.

Moreover, some studies have loaded non‐precious metals such as nickel (Ni) onto FE catalysts [124]. These single‐atom‐modified FE catalysts generally regulate the HER primarily through one of two mechanisms: (i) the phase transition between the FE and PE phases, which induces structural reorganization and consequently modulates H^+^ adsorption and H_2_ desorption at surface active sites; (ii) polarization switching, which drives reversible redistribution of surface charge density and thereby tunes the adsorption/desorption energetics of reaction sites. However, according to our survey, studies directly loading metals onto the surface of FE catalysts are relatively limited. Currently, a more common strategy is to construct heterostructures or introduce metal dopants into FE materials, as discussed in the next section.

#### 3.2.2 FE Heterostructure Examples: TiO_2_@BaTiO_3_/CdS and WSe_2_@In_2_Se_3_

As discussed in the introduction, solar‐driven photocatalytic water splitting for the HER using non‐precious metals has become a current research hotspot due to its potential for low cost and environmental friendliness. The key steps in photocatalytic water splitting include light absorption, separation of photogenerated charge carriers, and their migration to the surface to participate in the water splitting reactions of OER and HER. However, the practical application of this technology has long been constrained by the rapid recombination of photogenerated carriers [125]. Recent studies have shown that the built‐in electric field in FE materials can not only facilitate carrier separation but also effectively suppress their recombination, thus attracting extensive attention [78, 126]. However, charged particles in the electrolyte solutions can easily neutralize this built‐in electric field, thereby weakening its effect [127]. Therefore, effectively shielding these influences and maintaining a stable built‐in electric field to achieve high photocatalytic performance is of significant research importance.

**FIGURE 10:**
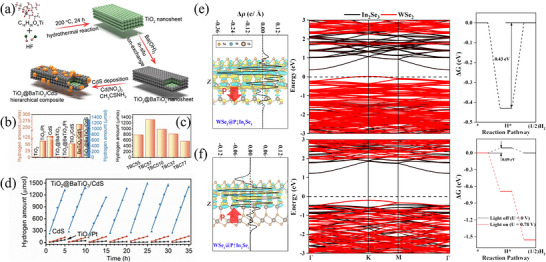
HER performance of FE heterostructure catalysts. (a) Schematic illustration of the synthetic process for nanosized CdS‐decorated TiO_2_@BaTiO_3_ nanocomposites. (b) Photocatalytic H_2_ production by pure TiO_2_, CdS and their composites after 5 h of photocatalysis. (c) Photocatalytic H_2_ production by TiO_2_@BaTiO_3_/CdS composites with different CdS contents. (d) Stability testing of TiO_2_/Pt, CdS, and TiO_2_@BaTiO_3_/CdS composite under light illumination [127]. Copyright 2022, Elsevier. The charge differential densities, electronic band structures, and Gibbs energy changes for the HER for In_2_Se_3_/WSe_2_ heterostructures with (e) downward (electrocatalytic behavior) and (f) upward (photocatalytic) polarization [85]. Copyright 2023, American Chemical Society.

Yang et al. [127] experimentally designed a sandwich‐structured heterojunction comprising a TiO_2_ core, a FE BaTiO_3_ shell, and a photosensitizing CdS layer (TiO_2_@BaTiO_3_/CdS) to investigate whether this configuration could effectively suppress the shielding effect of charged particles in electrolyte solutions on the built‐in electric field within the FE catalyst. Moreover, they synthesized seven single‐component and composite photocatalytic materials as the control group, including TiO_2_, CdS, TiO_2_/Pt, TiO_2_@BaTiO_3_, TiO_2_@BaTiO_3_/Pt, TiO_2_/CdS, and BaTiO_3_/CdS (Figure 10a). Subsequently, a five‐hour light irradiation experiment was conducted in the electrolyte solution, and H_2_ production was measured. The results indicated that TiO_2_, TiO_2_@BaTiO_3_, and TiO_2_@BaTiO_3_/Pt exhibited very low H_2_ production, while the H_2_ production of BaTiO_3_/CdS, TiO_2_/Pt, CdS, and TiO_2_/CdS was increased. Notably, the TiO_2_@BaTiO_3_/CdS composite photocatalytic material exhibited the highest H_2_ production, reaching 261.78 µmol (Figure 10b). They further investigated TiO_2_@BaTiO_3_/CdS composites with different ratios of the components, all of which exhibited high H_2_ production (Figure 10c). Moreover, they reported that cyclic photocatalytic tests on TiO_2_@BaTiO_3_/CdS revealed significantly superior stability compared to TiO_2_/Pt and CdS (Figure 10d). The TiO_2_@BaTiO_3_/CdS composite photocatalyst exhibiting the highest HER activity was attributed to the following two factors: First, its asymmetric architecture—characterized by differential interfacial contact areas and spectrally complementary light absorption—maintains the long‐term stability of the polarization electric field within BaTiO_3_. Second, this stable polarization field significantly suppresses the recombination of photogenerated charge carriers, thereby enhancing both the efficiency and durability of photocatalytic water splitting for H_2_ production. Collectively, this study proposes “FE polarization coupled with structural asymmetry” as a generalizable strategy to mitigate the screening effect of charged particles in electrolyte solutions on the built‐in electric field of FE catalysts. This strategy effectively suppresses the recombination of photogenerated charge carriers, thereby enhancing the photocatalytic efficiency of water splitting.

The screening effect of electrolyte solutions on polarization charges is a key physical characteristic that distinguishes liquid‐phase FE catalysis from solid‐state FE devices. In solid‐state environments, mobile liquid ions are absent, which allows for stable FE polarization states and significant polarization field effects. In contrast, at the solid‐liquid interface, a large number of free ions directly screen the surface polarization charges, thereby weakening the interfacial advantages conferred by polarization [127]. The above discussion on TiO_2_@BaTiO_3_/CdS composites only reflects the interfacial behavior of a single sample. In fact, the screening effect is regulated by multiple factors, including solution concentration, ion type, and solution pH, and represents a common bottleneck faced by all FE‐based liquid‐phase catalytic systems [99]. To date, while various strategies (e.g., interfacial engineering and structural engineering) have been explored to reduce the screening effect in FE catalysis, a unified understanding of the underlying mechanisms has yet to be established. Addressing this gap represents a key challenge that must be urgently tackled in future FE catalysis research.

Ju et al. [85] theoretically revealed that polarization‐dependent charge transfer in FE heterostructures can effectively regulate the electronic structure, allowing conversion between metallic behavior applicable for electrocatalysis and semiconducting behavior for photocatalysis. They validated the feasibility of Mo‐BN@In_2_Se_3_ for use in both photocatalytic and electrocatalytic systems based on the NRR. To verify the universality of this conclusion, they extended their research to the HER. Using WSe_2_@In_2_Se_3_ as another example (Figure 10e,f), they similarly found that the transformation between the metallic and semiconductor states could be achieved through FE polarization switching. Furthermore, they claimed that the activity of WSe_2_@In_2_Se_3_ in electrocatalytic HER primarily originates from Se vacancies, whereas its photocatalytic HER activity is governed by multiple factors, including light absorption capability, Se vacancies, and built‐in electric fields.

So far, numerous studies have focused on FE heterostructures and their application in (photo)catalysis for various reaction systems [29, 128, 129, 130]. In general, the catalytic performance of these FE heterostructures is co‐regulated by multiple factors, including the built‐in electric field, surface vacancies, *d*‐band center, and charge transfer.

### 3.3 Other Engineered FE Catalysts

As previously discussed in this perspective, FE materials can effectively suppress the rapid recombination of photogenerated electron–hole pairs in photocatalytic processes owing to their spontaneous polarization, thereby overcoming the efficiency limitations of traditional semiconductors. Meanwhile, the switchable nature of FE materials provides an important method of regulating the coupling between electrocatalysis and photocatalysis, as well as facilitating transformation among various catalytic reactions, such as HER, ORR, NRR, and CO_2_RR. In addition to strategies such as heterostructure engineering and single‐atom modification discussed above, strain engineering and doping can also significantly improve the efficiency of FE materials in catalyzing H_2_ production [131, 132, 133, 134]. These strategies will be discussed in this section, using BaTiO_3_ and BaTiO_3_@MoSe_2_ as representative examples.

Before reviewing the impact of strain engineering on the catalytic performance of FE catalysts, it is necessary to first clarify the potential correlation and differences between the piezoelectric effect and the FE effect. Specifically, all ferroelectrics are piezoelectric, but the converse is not true. Strain‐induced piezocatalysis relies on the piezoelectric potential generated by mechanical strain and does not require the switchable spontaneous polarization unique to ferroelectrics. Therefore, the two differ fundamentally in their core mechanisms and advantages. It is worth emphasizing that FE materials, as special materials possessing both piezoelectricity and ferroelectricity, can not only independently utilize the piezoelectric or FE effect, but also achieve synergistic coupling between piezoelectric strain catalysis and FE polarization catalysis. This is precisely the unique advantage that distinguishes FE‐based catalytic materials from ordinary piezoelectric materials [131].

**FIGURE 11:**
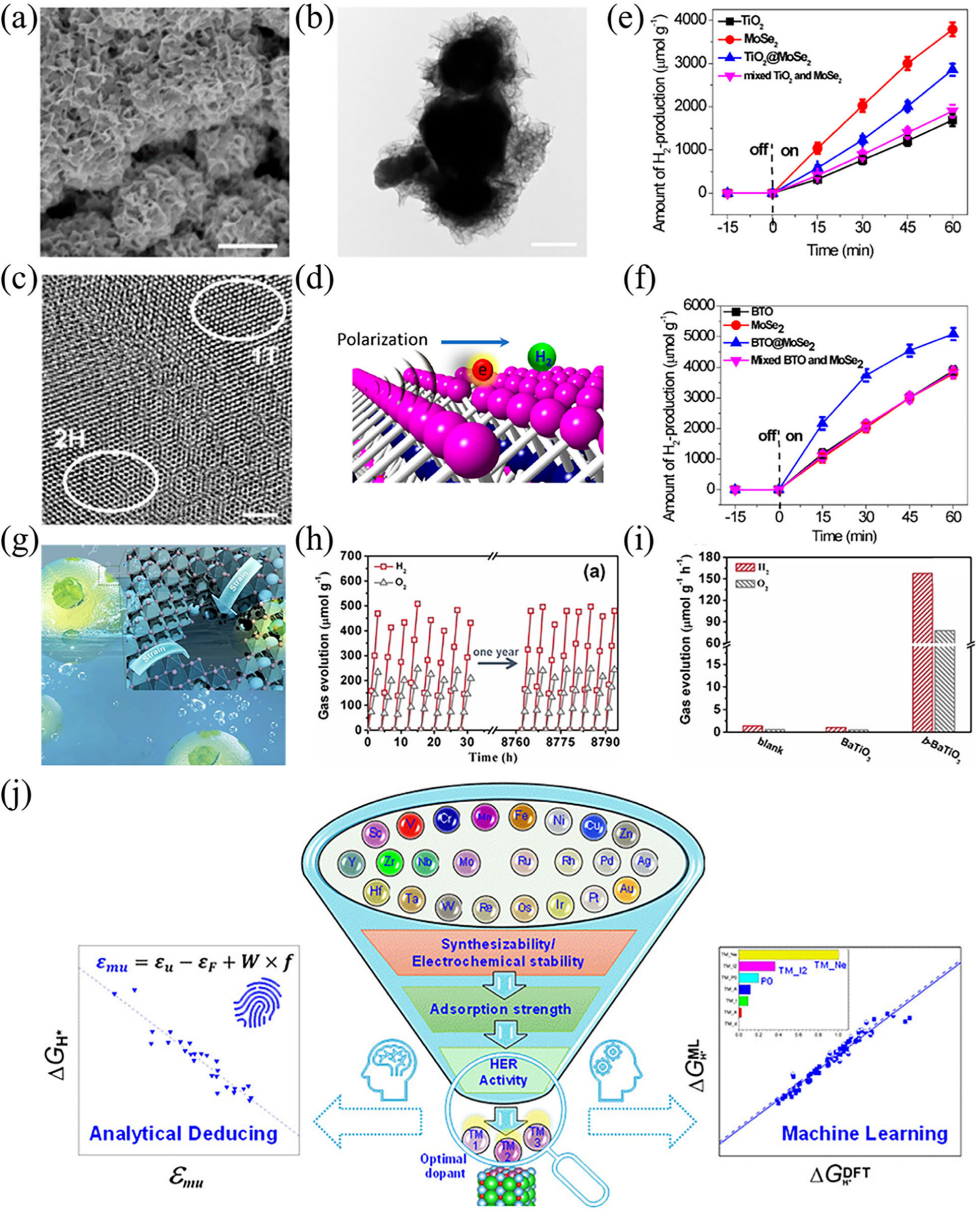
Other engineering regulation strategies (strain/doping) in FE catalysts. (a) Scanning electron microscope (SEM) image of BaTiO_3_@MoSe_2_ (Scale bar = 5 nm). (b) SEM image of BaTiO_3_@MoSe_2_ (Scale bar = 500 nm). (c) High‐resolution transmission electron microscopy (HR‐TEM) image of BaTiO_3_@MoSe_2_. (d) Schematic diagram illustrating how the HER activity of BaTiO_3_@MoSe_2_ is enhanced by the strain‐induced piezoelectric effect. H_2_ production under ultrasonic vibration and illumination for some pure single‐phase and composite materials (e) without and (f) with BaTiO_3_ [131]. Copyright 2021, American Chemical Society. (g) Schematic diagram illustrating how the HER activity of single‐phase porous FE catalyst BaTiO_3_ (denoted as *b*‐BaTiO_3_) is enhanced by the strain‐induced piezoelectric effect. (h) Cyclic tests of H_2_ and O_2_ production for *b*‐BaTiO_3_ under the piezoelectric effect. (i) H_2_ and O_2_ production rates of a blank sample, pure BaTiO_3_ nanoparticles and *b*‐BaTiO_3_ nanoparticles under 40 kHz ultrasonic vibration [132]. Copyright 2021, Wiley‐VCH GmbH. (j) Machine learning and theoretical calculations of transition metal‐doped FE BaTiO_3_ for the HER [133]. Copyright 2025, American Chemical Society.

To investigate the influence of strain effects in FE heterostructures on H_2_ production performance, Guo et al. [131] synthesized a series of single‐component and composite materials, including TiO_2_@MoSe_2_, MoSe_2_, TiO_2_, and BaTiO_3_@MoSe_2_. Scanning electron microscopy (SEM) and TEM analyses revealed that large amounts of monolayer or few‐layer MoSe_2_ were successfully loaded onto the surface of BaTiO_3_, with relatively large grain sizes (Figure 11a,b). Moreover, based on TEM results, they identified that the loaded MoSe_2_ exhibited two crystalline phases: the metallic 1T phase and the semiconducting 2H phase (Figure 11c). They proposed that the built‐in electric field of the FE BaTiO_3_, combined with externally applied mechanical strain, jointly induced a piezoelectric effect, thereby significantly enhancing the catalytic performance of BaTiO_3_@MoSe_2_ (Figure 11d). To further validate this mechanism, they employed ultrasonic vibration as an external strain source under light irradiation, using samples without BaTiO_3_ as controls. These results demonstrated that the H_2_ evolution rate of the BaTiO_3_@MoSe_2_ composite was significantly higher than those of all other materials compared (Figure 11e,f).

In investigating the influence of the piezoelectric effect on the H_2_ production performance of single‐phase BaTiO_3_, Su et al. [132] achieved controllable strain regulation in BaTiO_3_ by constructing a porous structure on the surface of BaTiO_3_ nanoparticles (Figure 11g). Under 40 kHz ultrasonic vibration, they systematically evaluated the water‐splitting performance of the single‐phase porous BaTiO_3_ nanoparticles. The results showed a H_2_ evolution rate of 159 µmol·g^−1^·h^−1^, and no significant decline in catalytic activity over a one‐year test, demonstrating excellent piezocatalytic stability (Figure 11h). To further validate the performance enhancement, they compared the material with pristine BaTiO_3_ and *g*‐BaTiO_3_ (gray BaTiO_3_ after surface oxidation), finding that the H_2_ production rate of the synthesized porous BaTiO_3_ nanoparticles increased by more than 100 times (Figure 11i). Based on the analysis of the underlying mechanism, they pointed out that the synthesized porous BaTiO_3_ structure provides favorable conditions for the introduction of strain. The strain applied through external vibration further enhances the polarization intensity of BaTiO_3_. Under this strain‐induced polarization enhancement, the piezoelectric potential of the porous structure is increased to 1.6 V, thereby compensating for the insufficient piezoelectric potential of pristine BaTiO_3_. Ultimately, the sustained presence of the polarization electric field effectively suppresses the recombination of photogenerated charge carriers, while the 1.6 V piezoelectric potential provides a sufficient driving force, resulting in excellent stability and durability of the HER.

Doping has been demonstrated to significantly enhance the catalytic performance of FE materials for various reactions. Qiu et al. [133] systematically investigated the effects of doping on HER performance under different polarization directions (including polarization upward (P↑), polarization downward (P↓), in‐plane polarization (P→)) based on a series of TM‐doped BaTiO_3_, using first‐principles calculations and machine learning approaches. They concluded that different polarization directions induce a reconstruction of surface charges, thereby modulating the catalytic activity of the TM atoms. Specifically, under P↑, the surface O atoms exhibit an electron‐deficient state, and the charge density of the TM atoms decreases accordingly. Under P↓, the surface O atoms become electron‐enriched, leading to an increase in the charge density of the TM atoms. In contrast, under P→, the surface charge distribution remains uniform. Furthermore, they found that the preferred adsorption site of H atoms varies with the type of TM atoms: for doping with *3d*‐TM atoms, H preferentially adsorbs at the O‐adjacent and Ba‐hollow sites, whereas for *4d*‐ and *5d*‐TM atoms, H tends to favor the TM‐top site. Interestingly, Mo‐doped BaTiO_3_ exhibits excellent catalytic activity across all FE polarization directions, including P↑, P↓, P→. The authors further proposed a novel electronic fingerprint based on the upper edge of the surface O 2*p*‐band before H adsorption as an activity descriptor. Additionally, through machine learning analysis, intrinsic correlations were revealed between the HER activity and surface‐independent properties, including the inherent characteristics of the TM dopant atoms and their polarization directions (Figure 11j).

In brief, both inducing the piezoelectric effect in FE catalytic systems through strain and adjusting the surface charges of FE materials by doping serve as effective approaches for enhancing the performance of the HER.

## 4 Summary and Outlook

At the conclusion of this perspective, we outline five forward‐looking directions for future research on FE materials in the context of catalysis of the HER.

First, the screening of polarization charges by free ions in electrolyte solution is a fundamental physical characteristic that distinguishes liquid‐phase FE catalysis from solid‐state FE devices. In solid‐state systems, the lack of mobile ions enables stable FE polarization and pronounced polarization‐field effects. In contrast, at the solid–liquid interface, abundant free ions directly screen surface polarization charges, substantially weakening the interfacial advantages arising from polarization. Furthermore, long‐term stability investigations of FE catalysts for the HER cycling test remain severely limited. More critically, current studies generally neglect the stability of FE polarization itself in the HER cycling test. As discussed above, polarization charges are readily screened by solution ions over time, which can undermine the interfacial advantages of polarization. Fortunately, various current strategies—such as interfacial and structural engineering—have been proposed to reduce this screening effect. Indeed, the screening of surface polarization charges is determined jointly by intrinsic material properties and extrinsic electrochemical variables—including electrolyte concentration, solution pH, and ionic speciation. Currently, most research focuses primarily on material modification. However, optimizing the ion type of the solution or adjusting its pH value may also effectively mitigate the screening effect faced by FE polarization.

Second, molecular FE catalysts offer a promising pathway to overcome the key application limitations of conventional FE catalysts in the field of H_2_ production [135]. Their advantages are mainly reflected in three aspects. First, molecular FE catalysts can be synthesized under mild, low‐temperature conditions, and most systems require no post‐polarization treatment, which substantially reduces production costs and energy consumption. Second, the excellent flexibility of molecular FE catalysts enables modulation of catalytic performance in HER systems. Third, benefiting from the reversible dissolution‐recrystallization characteristic, molecular FE catalysts can maintain stable catalytic activity and effectively reduce the polarization decay of conventional FE catalysts. Nevertheless, the T_C_ of most molecular FE catalysts is still lower than that of conventional counterparts. In this context, constructing heterostructures to integrate the two types of catalysts for complementary advantages and synergistic effects is expected to become a promising research direction in H_2_ production.

Third, as discussed in this perspective, most current FE materials are primarily concentrated in semiconductor or insulating systems, meaning their application in HER catalysis remains largely limited to photocatalysis. In fact, electrocatalysis continues to dominate current water‐splitting technologies. While previous studies have explored the application of FE materials in electrocatalytic HER through different strategies, including heterostructure construction, single‐atom modification, and other engineering approaches, intrinsic 2D FE metals (e.g., the MTe_2_ (M = Pt, Pd, Ni) family and PtBi_2_ with low precious‐metal content) [136, 137] may represent more promising and ideal candidate systems compared to these externally modulated methods. Moreover, compared with traditional FE semiconductor catalysts, FE metallic catalysts exhibit abundant physical effects owing to the emergence of electronic states near the Fermi level. Nontrivial topological states are a representative case in this regard. Recent advances in topological catalysis have revealed that topological surface states (e.g., Fermi arc or drumhead‐like surface states) can greatly enhance catalytic activity on material surfaces [23]. Taking metallic FE PtBi_2_ as an example, Yang et al. [137] confirmed that the nontrivial topological states near its Fermi level can generate robust Fermi arc surface states. Combined with surface polarization charges, these surface states are expected to effectively facilitate the HER. Moreover, since PtBi_2_ has been successfully synthesized in experiments [138], this material can serve as an ideal platform to experimentally verify the synergistic effect between topological states and polarization toward enhanced HER performance, which will represent a highly promising research direction.

Fourth, current research on FE catalysts is largely confined to traditional FE materials. However, with the rapid advancement of symmetry analysis and theoretical calculations, a wide array of unconventional FE systems has been discovered, including those exhibiting sliding ferroelectricity, type‐III multiferroicity, and fractional quantum ferroelectricity [102, 103, 104, 105, 106, 107, 108, 109, 139]. The application of these systems in water splitting remains scarcely reported. Particularly for type‐III multiferroicity, although various definitions exist within the research community, these materials in some way achieve coupling between ferroelectricity and magnetism. Studies on non‐polarized catalysts have demonstrated that spin can effectively enhance surface catalytic activity [140]. More importantly, for photocatalytic water splitting, the unique magnetoelectric coupling in type‐III multiferroic systems may enable synergistic enhancement of the two mechanisms rather than simple additivity. Traditional FE catalysts only improve charge transport via polarized electric fields but cannot regulate interfacial spin states, leading to disordered electron spins at active sites and sluggish reaction kinetics. In contrast, non‐polar magnetic catalysts can manipulate spin states but fail to utilize built‐in electric fields for charge separation, resulting in severe carrier loss. Through magnetoelectric interactions, dynamic synergy between polarized electric fields and spin ordering can be achieved: FE polarization governs charge transport, while spin ordering controls interfacial reaction kinetics and reaction pathways. This coupling may simultaneously address two core challenges in catalysis—low charge separation efficiency and slow interfacial reaction rates. We hope that this potential insight can provide experimenters with new ideas and be validated by experiments in the future.

Finally, the enhancement of catalytic reactions by topological surface states has increasingly attracted significant attention in the field of chemistry. Certain FE materials can undergo reversible transitions between topological and non‐topological states, as well as among distinct topological phases, during FE‐PE phase transitions [89, 141]. This characteristic enables FE materials to serve as a platform for investigating how different topological states influence surface catalytic performance through controlled topological phase transitions mediated by FE switching. Furthermore, whether the coupling between FE polarization and topological surface states can effectively enhance the HER performance, analogous to the piezoelectric effect [131, 132], represents a promising and important research direction.

Overall, this perspective provides a comprehensive examination of FE materials as catalysts for the HER.

## Author Contributions

**Sean Li**: investigation. **Zhenxiang Cheng**: writing – review and editing. **Weizhen Meng**: writing – review and editing, investigation, data curation, funding acquisition. **Wenxian Li**: writing – review and editing, conceptualization, project administration, supervision. **Rongxuan Lu**: writing – original draft, investigation, validation, formal analysis, data curation. **Michael Ferry**: investigation. **Judy N. Hart**: writing – review and editing, formal analysis.

## Conflicts of Interest

The authors declare no conflicts of interest.

## Data Availability

The data that support the findings of this study are available on request from the corresponding author. The data are not publicly available due to privacy or ethical restrictions.
